# Effect on skin hydration of using baby wipes to clean the napkin area of newborn babies: assessor-blinded randomised controlled equivalence trial

**DOI:** 10.1186/1471-2431-12-59

**Published:** 2012-06-01

**Authors:** Tina Lavender, Christine Furber, Malcolm Campbell, Suresh Victor, Ian Roberts, Carol Bedwell, Michael J Cork

**Affiliations:** 1 School of Nursing, Midwifery and Social Work, The University of Manchester, Manchester, UK; 2 School of Biomedicine, The University of Manchester, Manchester, UK; 3 Central Manchester NHS Foundation Trust, St Mary's Hospital, Oxford road, Manchester, UK; 4 Faculty of Life Sciences, The University of Manchester, Manchester, UK; 5 Academic unit of Dermatology Research, The University of Sheffield Medical School, Sheffield, UK

## Abstract

**Background:**

Some national guidelines recommend the use of water alone for napkin cleansing. Yet, there is a readiness, amongst many parents, to use baby wipes. Evidence from randomised controlled trials, of the effect of baby wipes on newborn skin integrity is lacking. We conducted a study to examine the hypothesis that the use of a specifically formulated cleansing wipe on the napkin area of newborn infants (<1 month) has an equivalent effect on skin hydration when compared with using cotton wool and water (usual care).

**Methods:**

A prospective, assessor-blinded, randomised controlled equivalence trial was conducted during 2010. Healthy, term babies (n = 280), recruited within 48 hours of birth, were randomly assigned to have their napkin area cleansed with an alcohol-free baby wipe (140 babies) or cotton wool and water (140 babies). Primary outcome was change in hydration from within 48 hours of birth to 4 weeks post-birth. Secondary outcomes comprised changes in trans-epidermal water loss, skin surface pH and erythema, presence of microbial skin contaminants/irritants at 4 weeks and napkin dermatitis reported by midwife at 4 weeks and mother during the 4 weeks.

**Results:**

Complete hydration data were obtained for 254 (90.7 %) babies. Wipes were shown to be equivalent to water and cotton wool in terms of skin hydration (intention-to-treat analysis: wipes 65.4 (SD 12.4) vs. water 63.5 (14.2), p = 0.47, 95 % CI -2.5 to 4.2; per protocol analysis: wipes 64.6 (12.4) vs. water 63.6 (14.3), p = 0.53, 95 % CI -2.4 to 4.2). No significant differences were found in the secondary outcomes, except for maternal-reported napkin dermatitis, which was higher in the water group (p = 0.025 for complete responses).

**Conclusions:**

Baby wipes had an equivalent effect on skin hydration when compared with cotton wool and water. We found no evidence of any adverse effects of using these wipes. These findings offer reassurance to parents who choose to use baby wipes and to health professionals who support their use.

**Trial registration:**

Current Controlled Trials ISRCTN86207019

## Background

There are major differences between the skin of a newborn infant and an older child or adult. The mean Trans Epidermal Water Loss (TEWL) has been shown to be 25 g/metre/hour in an infant compared with 7 in an adult (p <0.0005) [1]. Skin barrier development in babies remains incomplete until around 12 months of age [1]. This is important, as the thin skin barrier in an infant [2] makes it vulnerable to skin diseases such as atopic dermatitis and napkin dermatitis. It has been reported that around 20 % of babies develop atopic dermatitis [3,4] and 50 % develop napkin dermatitis [5]. These problems lead to concerns regarding skin care routines, for parents and health professionals.

A number of factors contribute to the development of napkin dermatitis. Prolonged skin contacts with urine and faeces, occlusion of the skin by napkin use, skin wetness and friction are key contributors. These factors cause a rise in skin surface pH, increasing the activity of proteases and lipases, thus hindering normal skin microflora and disrupting normal skin barrier integrity [6-9].

Whilst internationally it has been acknowledged that appropriate cleansing practices are important [10], a dearth of good quality clinical trials has led to variations in baby skin care regimens around the globe, influenced by tradition, culture and prior experience [10,11]. The guidelines produced by the US Association of Women’s Health, Obstetric and Neonatal Nurses recommend that detergent and alcohol free wipes may be used if a clean cloth and water are not available [12]. However, the Postnatal Care guidelines in the UK [13] recommend that wipes are not used for baby cleansing. Thus, in the UK, the use of cotton wool and water is recommended to parents.

Nevertheless, baby wipe usage has become progressively more common for the cleansing of newborn babies’ skin [14], despite the absence of good quality, controlled, clinical research tackling their safety and efficacy [14,15]. This may be because some parents do not have confidence in water alone as an effective cleanser [11]. One recent trial that did assess the impact of wipes versus water on skin integrity offered some reassurance of the safety of wipes but had limited applicability to healthy newborns as the study sample comprised of high risk neonates [16]. Trials that have compared wipes to water in healthy infants have been methodologically limited by not being sufficiently powered to detect clinically important differences [10,17]. Furthermore earlier trials have studied children greater than one month old; [14,17-20] this is relevant because of the ongoing development of babies’ skin, particularly in the first month of life [21]. Despite the limitations of these studies, all authors concluded that there was good skin tolerance of wipes and no evidence of harm, even when used on dermatitis skin [14].

Conversely, baby wipe-induced dermatitis in adults has also been documented [15,22] causing some health professionals to question the potential harm associated with the use of baby wipes and promote water use only [23].

However, water may not be an innocuous cleansing agent. The damaging effects of water in adults have previously been highlighted [24]. In babies, water is rapidly absorbed into the skin, even within 10 seconds [1]. This has the potential to disrupt the barrier function by increasing the space between skin cells. Furthermore, tap water has a pH between 7.9 and 8.2 [24] which is more alkaline than the pH of the skin in the weeks following birth. Skin integrity can be compromised if the acid milieu of the skin is altered [25].

At present, there is little data from clinical trials that can be used to assist parents and health professionals in choosing between water and cotton wool and an appropriately formulated baby wipe, for cleansing the buttocks for newborn healthy babies. This is an important issue because of the readiness to use baby wipes among mothers [11]. We therefore conducted a randomised controlled trial to test the hypothesis that the use of a cleansing wipe on the napkin area of newborn infants has an equivalent effect on skin hydration when compared with using cotton wool and water (usual care).

## Methods

### Study site and population

Between February and October 2010, we recruited healthy newborn babies delivered at a large teaching hospital in the North West of England, who were born at 37 weeks gestation or more and were using disposable nappies. Babies were excluded if they were admitted to the neonatal unit; were receiving phototherapy; had limb defects, non-traumatic impairment of epidermal integrity or evidence of skin disorder at first visit. Babies with a chromosomal abnormality or other syndromic diagnosis and babies going for adoption were also excluded.

The study followed Declaration of Helsinki protocols and received ethical approval from North West 11 Research Ethics Committee, Preston, [16^th^ October 2009] (REF: 09/H1016/118).

### Recruitment and randomization

Pregnant women of potentially eligible babies were supplied study information in the antenatal period via GP surgeries, and community and hospital clinics. In the postnatal period, the attending clinical midwives sought verbal permission for a research midwife to approach women who had been given prior information. Baseline details were collected from all women supplying verbal and written consent, using a self-administered questionnaire. Family history of atopic eczema was established at this stage (defined as at least one of father, mother, or sibling, who has had a medical diagnosis of atopic eczema and who has had topical steroid treatment). Interpreters were available for non-English speaking women.

Babies were randomized to a napkin cleansing regimen using a specific type of baby wipe or cotton wool and water. Randomisation was by computer-generated telephone randomization, set up by a Clinical Trials Unit. Randomization was stratified according to whether or not there was a family history of atopic eczema. The randomization sequence was in blocks of different sizes.

### Intervention

We chose the JOHNSON’S Baby Skincare Fragrance Free Wipe (Johnson & Johnson Ltd., Maidenhead SL6 3UG, UK), a product widely available in the market. The cleansing system contained: coco-glucoside and lauryl glucoside (non-ionic sugar derived surfactants). The emollients contained: glycerin and glyceryl oleate. The baby wipes also contained citric acid, which can have dual functionality as pH adjuster and chelator. Additionally, it was important to have a wipe with a pH close to the skin pH (around 4.9 in this case); if the pH is too low, this could be an irritant, if too high this would increase the protease activity and inhibit lipid lamellae synthesis in the skin barrier. The wipe contained 97 % water and was free of alcohol, fragrance, essential oils, soap and other harsh detergents; it was appropriately preserved to prevent growth of microorganisms. The wipe’s cloth material was a rayon viscose and polyester nonwoven fiber blend, entangled in a matrix trough water jets without chemical binders. This is designed to reduce friction when wiped across the skin surface.

Participating mothers were given a cleansing demonstration by a Health Care assistant. All mothers were advised to use nappies which were supplied by the researchers for the duration of the study to ensure similar absorbency; a factor likely to influence skin hydration. Mothers were also advised to avoid using napkin cream, other than that supplied by the research team as a rescue treatment. Parents were provided with cotton wool or baby wipes, according to their allocated trial arm.

### Assessment of trial outcomes

All initial measurements were taken in the hospital and follow up measurements were taken in the home. Our primary outcome measure was change in stratum corneum hydration scores on the buttocks from first assessment (within 48 hours of birth) to 4 weeks post birth, using a Corneometer [26].

Secondary outcomes comprised change in erythema measurements using a Mexameter (® MX 18) [27]; change in Trans Epidermal Water Loss (TEWL) using an Aquaflux (AF200) [28], and change in skin surface pH (using a pH meter). Measurements were taken on the babies’ buttocks at first assessment (within 48 hours of birth) and 4 weeks post-birth. Anatomical markers were used to ensure assessments were consistent. Two measurements were taken at each site; mean scores were used in analysis. Women were encouraged to cleanse the babies’ buttocks and replace the napkin 30 minutes before the assessments were conducted. If the baby soiled the napkin within the 30 minutes prior to proposed assessment, the assessment was delayed for a further 30 minutes. Swabs of the peri-anal area were also obtained at 4 weeks to observe for between-group differences in the presence of microbial skin contaminants and irritants (Coliform bacteria and Candida species). Skin measurement instruments were calibrated prior to recruitment, during the recruitment period and following the last follow-up assessment to prevent measurement error.

Clinical measures included napkin dermatitis observed at first assessment and 4 weeks post-birth by the research midwife, and napkin dermatitis observed by the mother daily from first assessment to 4 weeks post-birth. A semi-structured diary was provided to all mothers; this included a Diaper Area Rash grading scale [29,30] to aid the process. A set of reference photographs depicting the various levels of diaper rash were used alongside the scale to ensure consistency in clinical observations. Nappy cream usage was also recorded and a rescue treatment was pre-specified (Natusan Nappy Cream). Creams were weighed at the outset of the study and following the last assessment as an accurate measure of usage.

### Blinding

All measurements were made by research midwives blind to treatment allocation. Women were asked not to reveal their allocation to the midwife assessing the baby in their home. Women were telephoned by the recruiting midwife on the day of assessment and reminded to remove visible signs of products (e.g. wipes or cotton wool) prior to the assessing midwife’s arrival. When the baby had a soiled nappy immediately prior to or during the assessment, the assessing midwife left the room until the baby had been cleaned.

### Compliance

To optimize levels of compliance we maintained weekly telephone contact with participating women; asked participants to keep empty wipes and cotton wool packets to determine usage; requested participants keep a cleansing activity diary; and supplied all wipes, cotton wool, napkin cream and disposable nappies for the first 4 weeks of the study. We pre-specified that we would assess the impact of any non-compliance on the primary outcome using three definitions. *Strict compliance* was defined as the non-use of any additional products on the test site areas (or areas likely to contaminate the test areas) throughout the study. *Mostly compliant* was defined as no more than two occasions of additional product use and non-use of additional products during the week of follow up assessment. *Non- compliance* was more than two occasions of additional product use and/or the use of additional products in the assessment week.

### Analysis

Data were analyzed using SPSS (Release 16), following double-entry of all data, with the statistician blinded to the true treatment allocation.

The primary analysis was a comparison of stratum corneum hydration score on the buttocks at 4 weeks between groups adjusted for the corresponding initial score using analysis of covariance, with the stratification variable (family history of atopic eczema) included as a factor. A 95 % confidence interval for the difference between the groups in adjusted mean scores was compared with the pre-determined equivalence region of -5.8 to +5.8 to determine whether the interventions were equivalent or not [31]. The main analysis was by intention-to-treat (ITT); pre-planned secondary analyses examined the effect of different protocol violations a per-protocol approach in case violations brought outcome scores for the water group closer to those for the wipes group and increased the chance of a Type I error (wrongly concluding equivalence) under ITT [31].

Secondary outcomes of skin pH, TEWL and erythema at the buttocks were also compared by group at 4 weeks using analysis of covariance to adjust for initial scores, with family history of atopic eczema as a factor. These were not equivalence analyses, and ITT was used for a conservative analysis. Other secondary outcomes were compared by randomized group under ITT: levels of Coliform bacteria and Candida species in the peri-anal area at 4 weeks using the Mann-Whitney test (distributions were highly skewed); midwife-observed napkin dermatitis (5-point scale) at 4 weeks using the chi-square test for trend; and maternal-observed napkin dermatitis total score at 4 weeks from diaries (sum of 28 5-point scores) using the Mann-Whitney U test. Kendall’s tau_b_ correlation was used to measure the association between highly skewed variables. Throughout, a two-tailed p ≤ 0.05 was considered to be statistically significant.

### Sample size

From our best available data on newborns [32], the mean stratum corneum hydration score at the thigh at 4 weeks post-birth was 58.0 (SD 14.9). We hypothesized an equivalence region of ±10 %, -5.8 to +5.8, for the difference in mean scores in the two groups. For one-sided 2.5 % significance in each tail to give a 95 % confidence interval for this difference, 140 per group were needed at trial end. Because these data were not collected from the buttocks, an internal pilot was overseen by our Data Monitoring Committee to confirm the accuracy of the trial size. Across 29 infants in the two groups, the pooled standard deviation for hydration at the buttocks at 4 weeks was 12.0; to allow for underestimation in a small sample, the upper 80 % confidence limit for this was estimated as 13.7. For the same equivalence region of -5.8 to +5.8, with one-sided 2.5 % significance in each tail to give a 95 % confidence interval for the difference in means between the groups [33] at 80 % power, we required 119 participants per group at trial end. Assuming a 10 % drop-out over 4 weeks, we aimed to recruit at least 133 participants per group at baseline.

## Results

**Figure 1:**
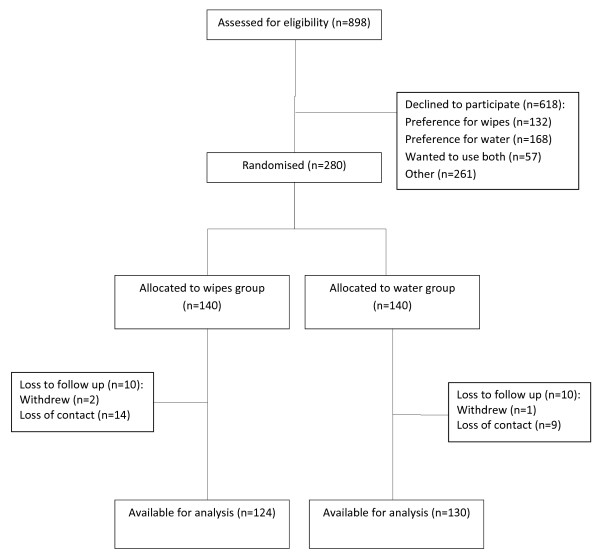
Participant flow during the study.

Overall 280 babies were randomized, equally between groups. The participation rate was 31.2 % (280/898). All participants received their randomly allocated intervention. The overall loss to first follow-up was 26/280 (9.3 %); 16 (11.4 %) in the wipes group and 10 (7.1 %) in the water group. Participant flow can be seen in Figure 1. One participant in the wipes group and two in the water group discontinued their allocated intervention by first follow-up. By first follow up, 107/124 participants in the wipes group were strictly compliant with the study protocol, 8 were mostly compliant and 9 not compliant. In the water group, 111/130 participants were strictly compliant, 10 were mostly compliant, 8 were not compliant and 1 was difficult to determine. The groups demonstrated similar baseline characteristics (Table 1). All initial skin assessment measures were also comparable by randomized group (Table 2). Babies in the wipes group had more frequent nappy changes than those in the water group (wipes: 54/123 had nappy changes at least every two hours (43.9 %); water: 40/129 (31.0 %), χ^2^_TREND_ = 4.83, df = 1, p = 0.028), but frequency of bathing was the same in the two groups (wipes: median number of baths per week = 3, range 0 to 14; water : median = 4, range 1 to 14, Mann-Whitney U = 7379.0, p = 0.32). There was no significant difference between the groups in the time interval between last nappy change and assessment (wipes: median = 30 minutes, range 10 to 325; water: median = 30 minutes, range 0 to 205, Mann-Whitney U = 7511.0, p = 0.53). There were no reported incidences of diarrhoeal illness during the study.

**Table 1:** Baseline characteristics of participants by randomised group. Values are numbers (percentages) unless stated otherwise

**Characteristic**	**Details**	**Wipes**	**Water**
		**(n = 124)**	**(n = 130)**
Family history of atopic eczema	Yes	44 (35.2)	49 (37.7)
Maternal age	Mean (SD)	28.6 (5.5)	29.5 (5.5)
Maternal ethnicity	White British	64 (51.6)	64 (49.2)
	White other	7 (5.6)	7 (5.4)
	Asian	25 (20.2)	25 (19.2)
	Black	24 (19.4)	21 (16.2)
	Other	4 (3.2)	13 (10.1)
Parity	Primiparous	55 (44.4)	68 (52.3)
	Multiparous	69 (55.6)	62 (47.7)
Birth mode	Normal vaginal	84 (67.7)	88 (67.7)
	Instrumental	25 (20.1)	22 (16.9)
	Caesarean section	15 (12.1)	20 (15.4)
Baby’s gender	Male	76 (61.3)	75 (57.7)
	Female	48 (38.7)	55 (42.3)
Birth weight	Mean (SD)	3.404 (0.5)	3.462 (0.5)
Feeding method	Breast	91 (73.4)	95 (73.1)
	Bottle	20 (16.1)	23 (17.7)
	Mixed	13 (10.5)	12 (9.2)

**Table 2:** First skin assessments by randomised group

**Measurement**	**Wipes (n**^**1**^ **= 124)**	**Water (n**^**1**^ **= 130)**
Hydration (arbitrary units)	40.0 (12.6)	38.8 (12.3)
Erythema (arbitrary units)	572.0 (104.2)	592.4 (105.1)
Log erythema (arbitrary units)	6.3 (0.2)	6.4 (0.2)
Skin pH	6.0 (0.6 %)	5.9 (0.6 %)
TEWL (g/m2/h )	12.4 (4.0 %)	13.0 (4.1 %)

^1^ occasional missing values due to equipment malfunction.

### Primary analysis

**Figure 2:**
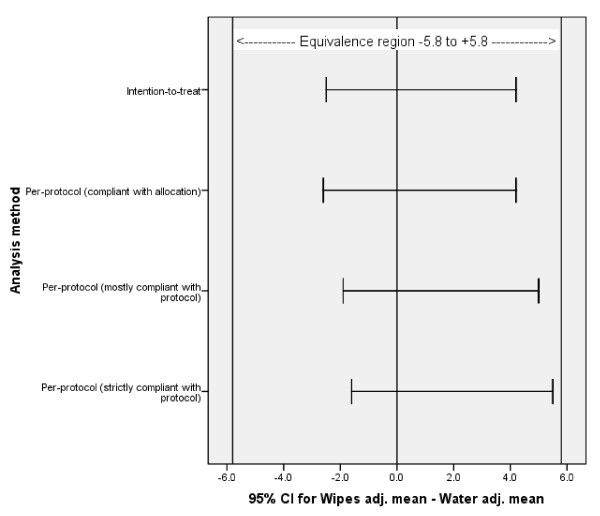
Equivalence analysis by confidence interval for difference in mean hydration score at 4 weeks between groups.

Mean hydration scores in the wipes group were similar in all four analyses (Table 3). Mean scores in water group were also similar, suggesting that non-compliance was not affecting the hydration score. There was no evidence of association between baseline hydration score and group. Wipes were shown to be equivalent to water and cotton wool in terms of skin hydration (Intention-to-treat analysis: wipes 65.4 (SD 12.4) vs. water 63.5 (14.2), p = 0.47, 95 % CI for difference between adjusted means -2.5 to 4.2; per protocol analysis: wipes 64.6 (12.4) vs. water 63.6 (14.3, p = 0.53, 95 % CI -2.4 to 4.2). Ninety-five percent confidence intervals for all four differences lay within the pre-specified equivalence region from -5.8 to 5.8 (Figure 2). Follow-up was intended to be at 28 days, but 32 follow-up skin assessments were carried out from 32-38 days with another 10 at 39 days or more, due to maternal availability. Per-protocol analyses examining the impact of excluding one or both subgroups also found no differences between the groups, with confidence intervals within the equivalence region.

**Table 3:** Comparison of primary outcome measure (hydration score on the buttocks) at follow-up by randomised group under intention-to-treat (ITT) and per-protocol (PP) analyses

**Analysis**	**Wipes (n = 123) Mean (SD)**	**Water (n = 126) Mean (SD)**	**Group effect P-value**	**95 % CI for difference in adjusted means**
ITT	65.4 (12.4)	63.5 (14.2)	0.470	−2.5 to 4.2
PP				
Compliant with allocation	(n = 122) 64.6 (12.4)	(n = 123) 63.6 (14.3)	0.526	−2.6 to 4.2
Mostly compliant with protocol	(n = 114) 64.9 (12.5)	(n = 116) 63.2 (14.1)	0.427	−1.9 to 5.0
Strictly compliant with protocol	(n = 106) 65.3 (12.0)	(n = 107) 63.2 (14.1)	0.440	−1.6 to 5.5

### Secondary analyses

Table 4 presents the secondary outcomes. We found no evidence of difference in means between groups in skin surface pH [5.93 (SD 0.58) vs. 5.65 (0.62), p = 0.36, 95 % CI -0.1 to 0.4], TEWL [17.8 (7.0) vs. 19.0 (10.6), p = 0.49, 95 % CI -3.9 to 1.2] or log erythema [6.27 (0.14) vs. 6.30 (0.14), p = 0.18, 95 % CI -0.06 to 0.01].

**Table 4:** Comparison of secondary outcomes at follow-up by randomised group under intention-to-treat analyses

**Analysis (ITT)**	**Wipes (n = 124)**	**Water (n = 130)**	**Group effect P-value**	**95 % CI for difference in adjusted means**
pH^1^, mean (SD)	(N = 118) 5.93 (0.58)	(N = 122) 5.65 (0.62)	0.356	−0.1 to 0.4
TEWL^1^, mean (SD)	(n = 119) 17.8 (7.0)	(N = 124) 19.0 (10.6)	0.486	−3.9 to 1.2
Log erythema^1^, mean (SD)	(n = 118) 6.27 (0.14)	(n = 129) 6.30 (0.14)	0.177	−0.06 to 0.001
Coliform bacteria^2^, median (IQR)	(n = 122) 2000 (53 to 45250)	(n = 128) 4850 (168 to 119500)	0.913	-
Candida species^2^, median (IQR)	0 (0 to 0)	0 (0 to 0)	0.454	-
Mother-reported dermatitis total 4-week score				
Complete responses, Median (IQR)	(n = 93) 1.00 (0 to 7.5)	(n = 96) 2.5 (0 to 12.75)	0.025	-
Partial responses, Median (IQR)	(n = 108) 1.00 (0 to 7)	(n = 116) 2.33 (0 to 11.8)	0.016	-
Midwife-reported dermatitis at week 4, n (%)			0.300	-
No evidence of rash	108 (87.1)	109 (83.8)		
Slight rash	13 (10.5)	14 (10.8)		
Mild-moderate rash	2 (1.6)	5 (3.8)		
Moderate-severe rash	1 (0.8)	2 (1.5)		

^1^Buttocks.

^2^Peri-anal area.

There were few reports of napkin dermatitis by mothers (over all 4-week diary entries, a complete absence of dermatitis was reported for 84 (37.5 %) babies [49 wipes v 35 water]), or the midwives (at follow-up, 217 (85.4 %) babies were judged to have no rash and 27 (10.6 %) a slight rash). We did detect evidence of difference in napkin dermatitis scores between groups, as reported daily by the mothers. Mothers of babies in the wipes group were less likely to report napkin dermatitis than those in the water group. This difference remained regardless of whether we compared those diaries that had complete entries (i.e. daily entries) (p = 0.025), or those who had incomplete entries (i.e. some days missing) (p = 0.016). We found no significant difference in napkin dermatitis scores between groups from the midwife’s single observation. Agreement between the mother and the midwife at follow-up appeared to be good. Among the 72 mothers who were followed up just as their diary ended, 57 (79.2 %) had 28-day scores that agreed with the midwife’s score, 6 scored one grade higher and 6 scored one grade lower. Among the 119 followed up within a day of their diary ending, 94 (79.0 %) agreed with the midwife, with 13 one grade higher and 8 one grade lower. Among the 147 followed up within two days of their diary ending, 114 (77.6 %) agreed with the midwife, with 18 one grade higher and 8 one grade lower.

The time from the last napkin change to the follow-up skin assessment was not correlated with the number of colonies of enteric Coliform bacteria (Kendall’s tau_b_ = 0.04, p = 0.37) or Candida species (Kendall’s tau_b_ = -0.04, p = 0.50), although the bacteria counts were themselves correlated (Kendall’s tau_b_ = 0.16, p = 0.002). We found no evidence of difference between groups in the numbers of Coliform bacteria [20000 (53-45250) vs. 4850 (168 to 119,500), p = 0.91] or Candida species [0 (0 to 0) vs. 0 (0 to 0), p = 0.18], in the peri-anal area. There was also no evidence of difference in use of Natusan cream (rescue napkin cream).

## Discussion

When products such as baby wipes appear on the shelves for purchase, an assumption is made that they have been evaluated for safety and efficacy [11]. While these aspects can be established through routine pre-market evaluation and meet global regulatory standards, many products do not undergo further clinical research to assess the effects of the products on the infants’ skin, as we have reported. In our study, we adhered to the same stringent guidelines as those recommended by the U.K. Medicines and Healthcare products Regulatory Agency [33] believing, like others [34], that products used on babies should be subjected to the same gold standard that is recognised for the use of medicines.

We report findings from the largest clinical trial of healthy newborn babies and napkin cleansing. Using specially formulated baby wipes had an equivalent affect on hydration of the babies’ buttocks as using water and cotton wool. Furthermore there was no evidence of any differences between the two cleansing regimens for any of the additional skin assessments (skin surface pH, erythema, trans epidermal water loss or microbial skin contaminants). This should reassure parents who choose to use baby wipes of a similar formulation to those used in this trial and to health professionals who support their use.

Wipes contain different ingredients and have changed notably over the last decade; the introduction of pH-buffered wipes, in particular, has improved napkin area care [35]. Those associated with contact dermatitis are likely to be older formulations which contain alcohol, sub-optimal surfactants and non-allergy screened fragrances. However, we only trialled one brand of wipe; given the plethora of wipes available, with different formulations, it would be important to conduct further robust randomized controlled trials before any widespread recommendation.

Although reports of severe napkin rash were few, there were more reports of mild/moderate napkin dermatitis by the mothers in the water group than in the wipes group. There were no differences between the groups in the midwives reporting. Whilst intuitively one may give more credence to the midwives score, she only assessed the babies’ buttocks at two time periods, four weeks apart. Conversely, the mother assessed the baby daily and was most likely to know the minutia of their own baby’s skin; the mother could therefore be seen as the expert. The value of parental assessment has been reported in atopic eczema where measurements made by the patient/carer using POEM correlated very well with clinical measurements made by the dermatologists [36]. The midwife was blind to the allocation but the mother was not. This may have influenced reporting; however, there is no clear rationale for why mothers in the usual care group (water) would report more negatively, especially as compliance was good. Napkin dermatitis was a secondary outcome and therefore the findings should be viewed cautiously. However, napkin dermatitis is the most common dermatologic disorder of infancy [37]; given the anxiety that this creates for parents, this is an area that warrants further investigation.

The absence of robust evidence from randomized controlled trials has meant that traditional practices and consensus opinions have dictated cleansing practices [10,11]. Our findings should be used to provide parents and health professionals with evidence based information, from which they can select the most appropriate method of napkin cleansing.

Our findings add to current debates [11,34] regarding the most appropriate cleansing regimens for babies. Uniquely, we have conducted the only adequately powered trial comparing wipes with cotton wool and water on healthy newborn infants. Importantly, our trial had a pragmatic design, which enabled us to assess the impact of wipe usage by those who normally use them (i.e. parents), in an environment that they are normally used. This latter point is particularly relevant given the mobility of wipes and the potential storage options (e.g. shelves, handbags, perambulators). We did, however, only access women in one hospital setting. Nevertheless, this setting supports women from a diverse range of socio-cultural backgrounds and our data reflect this. Findings from this study are therefore likely to be transferable to other settings.

Our trial observed outcomes at 4 weeks post birth, providing information on the impact of water and one type of wipe on skin integrity in the early postpartum period. Longitudinal studies would be required to assess the impact of these cleansing regimes with continued use. We do not know whether wipes using other formulations would have comparable results to ours; further trials are required.

## Conclusions

Our study is the largest clinical trial of healthy newborn babies and napkin cleansing. We provide valuable data which demonstrated that using specially formulated baby wipes had an equivalent affect on hydration of the babies’ buttocks as using water and cotton wool. These findings should be used by parents when choosing their preferred napkin cleansing routine. Health professionals should use this information to support parental choice.

## Competing interests

All authors have completed the Unified Competing Interest form at http://www.icmje.org/coi_disclosure.pdf (available on request from the corresponding author) (URL) and declare: financial support for the submitted work from Johnson and Johnson; TL, CF, CB, SV, MJC, IR have received a research grant for this submitted work and other ongoing research (TL, CB, MJC). TL, MJC, CB have received honorariums for acting as expert advisors to Johnson and Johnson.

This study was funded by Johnson and Johnson, however, this is an investigator led trial; the trial management team (TL, MJC, CB, SV, IR, CF), made all decisions regarding trial design, execution, analysis, interpretation and publication. Only the trial management team and data monitoring committee had access to the data.

## Authors’ contributions

TL and MJC conceived the idea for the study. TL, MJC, MC, CF, CB, SR, IR designed the study. CF co-ordinated the data collection. MC conducted the analysis. All authors interpreted the findings. TL wrote the first draft of the paper. All authors approved the final draft of the paper. TL is guarantor of the paper.

## Funding

This is an industry funded study, however the trial was investigator led. The trial management team ran the study independently of the funders, from conception to completion; this included trial design, management, data analysis, interpretation of results and production of this manuscript. The funders have not had access to the actual data.

## Pre-publication history

The pre-publication history for this paper can be accessed here:

http://www.biomedcentral.com/1471-2431/12/59/prepub
